# Unravelling the Complex Denaturant and Thermal-Induced Unfolding Equilibria of Human Phenylalanine Hydroxylase

**DOI:** 10.3390/ijms22126539

**Published:** 2021-06-18

**Authors:** María Conde-Giménez, Javier Sancho

**Affiliations:** 1 Departamento de Bioquímica y Biología Molecular y Celular, Biocomputation and Complex Systems Physics Institute (BIFI)-Joint Units: BIFI-IQFR (CSIC) and GBsC-CSIC, University of Zaragoza, 50009 Zaragoza, Spain; mcondeg@unizar.es; 2 Aragon Health Research Institute (IIS Aragón), 50009 Zaragoza, Spain

**Keywords:** phenylalanine hydroxylase, phenylketonuria, protein stability, protein folding, pharmacological chaperone

## Abstract

Human phenylalanine hydroxylase (PAH) is a metabolic enzyme involved in the catabolism of L-Phe in liver. Loss of conformational stability and decreased enzymatic activity in PAH variants result in the autosomal recessive disorder phenylketonuria (PKU), characterized by developmental and psychological problems if not treated early. One current therapeutic approach to treat PKU is based on pharmacological chaperones (PCs), small molecules that can displace the folding equilibrium of unstable PAH variants toward the native state, thereby rescuing the physiological function of the enzyme. Understanding the PAH folding equilibrium is essential to develop new PCs for different forms of the disease. We investigate here the urea and the thermal-induced denaturation of full-length PAH and of a truncated form lacking the regulatory and the tetramerization domains. For either protein construction, two distinct transitions are seen in chemical denaturation followed by fluorescence emission, indicating the accumulation of equilibrium unfolding intermediates where the catalytic domains are partly unfolded and dissociated from each other. According to analytical centrifugation, the chemical denaturation intermediates of either construction are not well-defined species but highly polydisperse ensembles of protein aggregates. On the other hand, each protein construction similarly shows two transitions in thermal denaturation measured by fluorescence or differential scanning calorimetry, also indicating the accumulation of equilibrium unfolding intermediates. The similar temperatures of mid denaturation of the two constructions, together with their apparent lack of response to protein concentration, indicate the catalytic domains are unfolded in the full-length PAH thermal intermediate, where they remain associated. That the catalytic domain unfolds in the first thermal transition is relevant for the choice of PCs identified in high throughput screening of chemical libraries using differential scanning fluorimetry.

## 1. Introduction

**Figure 1:**
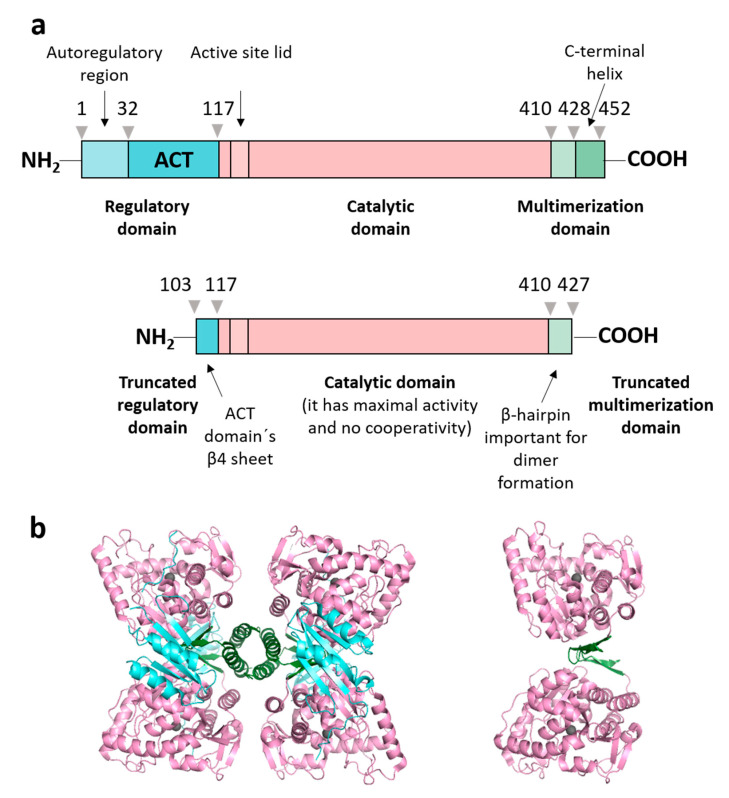
Human PAH domain structure and constructions used. (**a**) Schematic representation of the full-length PAH construction (residues Met 1-Lys 452, named PAHt) and of a double truncated form (residues Gly 103-Thr 427, named PAHd) with annotated structural and functional domains: regulatory domain (in aquamarine), catalytic domain (in light pink) and multimerization domain (in green). The truncated form, PAHd, lacks most of the regulatory and multimerization domains. (**b**) Structures of the two PAH constructions: tetrameric PAHt (left) and dimeric PAHd (right) forms using as templates the crystal structures of the full-length human PAH in the resting state (mutant C29S, PDB 6N1K) [13] and the double truncated human PAH form (ΔN1-102/ΔC428-452) (PDB 1J8T) [14]. Note that residues 103–117 are missing in the 1J8T PDB file.

Human phenylalanine hydroxylase (PAH) (EC 1.14.16.1) is a homotetrameric enzyme (Figure 1) that catalyzes the hydroxylation of L-phenylalanine to produce L-tyrosine in liver. It is a pterin-dependent enzyme, which needs iron and molecular dioxygen for activity [1]. Some genetic variants of the gene encoding this enzyme cause the rare autosomal recessive disorder called phenylketonuria (PKU) [2]. PAH variants are compiled in the Phenylalanine Hydroxylase Gene Locus-Specific Database (*PAH*vdb, http://www.biopku.org, accessed on 16 June 2021). There are more than 700 disease-associated missense variants distributed throughout the three structural domains of the protein [3]. Most of them induce loss of conformational stability and decreased physiological enzymatic activity characterized, if it is untreated, with increased Phe blood levels and toxic Phe brain levels. The equilibrium among different tetrameric conformers, with different intrinsic levels of activity, has been proposed to be altered in some disease-associated variants [4].

Kuvan, a synthetic form of the tetrahydrobiopterin PAH cofactor, was the first approved drug treatment for PKU patients with a milder form of the disease [5]. One of Kuvan’s proposed mechanism of action is a chaperoning effect in the enzyme folding equilibrium [6,7]. Pharmacological chaperones act by binding to the native state of an unstable enzyme variant, displacing the folding equilibrium, as per law of mass action, toward the native state, thereby rescuing its physiological function [8]. A deeper understanding of the PAH folding equilibrium could help to develop new useful chaperone-based therapeutic approaches.

Characterization of the folding equilibrium of a multidomain homomultimeric enzyme such as PAH, where both dissociation of subunits and full or partial unfolding of individual domains can take place, is not a minor task. While the study of protein stability issues using computational approaches such as Molecular Dynamics simulation has become feasible [9,10], a full description of the unfolding equilibrium of fairly large proteins as a function of denaturant and temperature is still best done through experiment. In previous biophysical work done on full-length PAH [11,12], the urea and thermal-induced PAH unfolding equilibria were studied and two transitions were observed in each case. A gradual dissociation of the native tetramer to increasingly denatured dimers and monomers was proposed to occur during urea denaturation. Furthermore, conformational changes in the C-terminal helix were suggested to cause the shift in oligomeric species associated with the first transition [11]. For thermal-induced PAH denaturation, a three-step model was proposed: first, the four regulatory domains unfold then two of the four catalytic domains unfold and finally, at higher temperature, irreversible protein denaturation accompanied with formation of protein aggregates occurs [12].

We studied the urea-induced and the thermal denaturation of full-length recombinant human PAH and of a truncated form lacking the regulatory domain and the C-terminal tetramerization helix (Figure 1). Spectroscopic analyses along with calorimetric and analytical ultracentrifugation data allowed us to propose a qualitative model for the distribution of the different species in the unfolding equilibria in which the catalytic domains, rather than the regulatory ones, appeared to protagonize the initial unfolding events. Analytical ultracentrifugation analysis performed on the chemical intermediates revealed that, rather than being well-defined species, they are polydisperse ensembles constituted by aggregates of different size.

## 2. Results and Discussion

### 2.1. Fluorescence Characterization of Urea-Induced Denaturation

**Figure 2:**
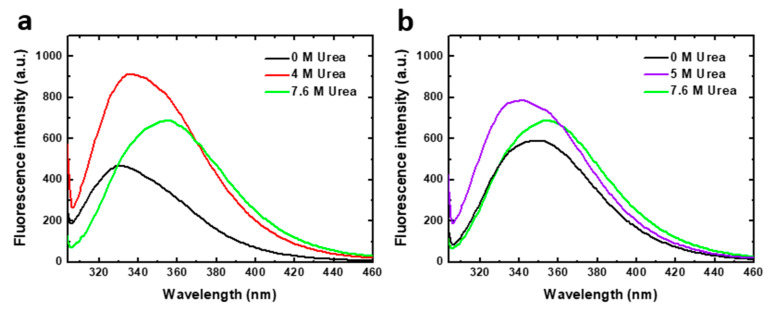
Tryptophan intrinsic fluorescence of human PAH in native and denaturing conditions. Emission spectra (excitation at 295 nm) of (**a**) 8 µM PAHt (full-length construction) and (**b**) 8 µM PAHd (double truncated construction) in 0, 4, 5 and 7.6 M urea.

**Figure 3:**
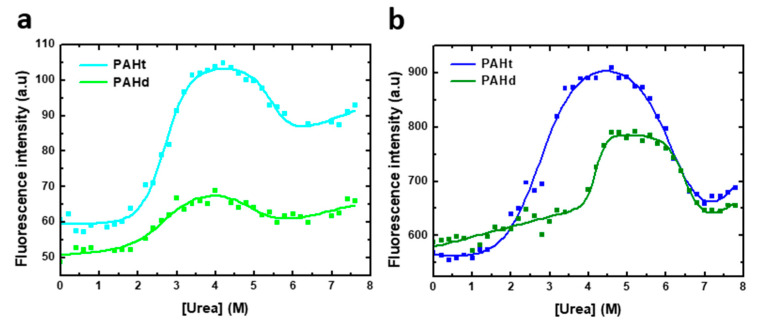
Urea-induced denaturation of human PAH protein at different protein concentrations. Denaturation was monitored by fluorescence intensity at 345 nm (excitation at 295 nm) of (**a**) 0.8 µM of full-length PAHt construction and truncated PAHd construction and (**b**) 8 µM of PAHt and PAHd. Continuous lines correspond to the nonlinear fitting curve obtained by using a three-state folding model.

Chemical denaturation was followed by fluorescence spectroscopy. Tryptophan fluorescence emission spectra of the two human PAH constructions analyzed, obtained at representative urea concentrations of the denaturation equilibrium, are shown in Figure 2. PAH contains three tryptophan residues (Trp120, Trp187 and Trp326) of which the major contributor to fluorescence intensity in native [15] and denaturing conditions has been reported to be Trp 120 [11]. In native conditions, in the absence of urea, the PAHt fluorescence spectrum shows an emission maximum at 330 nm. As the protein unfolds, the maximum shifts to higher wavelengths indicating that the Trp residues become more exposed to solvent. In 4 M urea (the denaturant concentration of maximal accumulation of an unfolding intermediate observed at the same protein concentration, see Figure 3b) the maximum appears red-shifted by 5 nm, and the intensity is largely increased. Increases in urea concentrations are known to moderately increase the quantum yield of Trp residues [16]. However, such a large fluorescence increase observed at 4 M urea cannot be attributed solely to the effect of urea. It seems that the fluorescence quantum yield of Trp residues in the native state of PAH is quenched by internal interactions and that, in the intermediate state, the quenching disappears or becomes less pronounced. In 7.6 M urea, (the denaturant concentration where the protein appears to be unfolded at the same protein concentration, see Figure 3b), a further red-shift of 20 nm can be observed that brings the maximum to 355 nm, a characteristic wavelength of maximal emission for fully solvent exposed Trp residues [17]. The decreased in quantum yield at 7.6 M urea relative to that at 4 M urea is opposed to the expected effect of urea on Trp emission, indicating that new interactions are established that quench the emission.

The emission spectrum of truncated PAHd in native conditions (0 M urea) displays a maximum at about 347 nm, clearly indicating that in this construction the tryptophan residues are much more exposed to solvent in native conditions than in full length PAHt. In 5 M urea (where maximal accumulation of an unfolding intermediate at the same protein concentration was observed, see Figure 3b) the maximum appears blue-shifted by 7 nm, and the intensity is also increased. In 7.6 M urea (the denaturant concentration where PAHd appears to be unfolded at the same protein concentration, see Figure 3b) the occurrence of the maximal emission at 356 nm is also indicative of full exposure of the tryptophan residues to solvent.

The different emission maxima exhibited at 0 M urea by the full-length and the truncated PAH forms is noteworthy. The truncated form, PAHd, shows the same tryptophan emission maximum as the full–length enzyme when it has been activated by its substrate L-Phe [15,18]. Substrate activation has been described to induce a conformational change in the enzyme that is evidenced by fluorescence intensity emission changes [15,19,20,21]. PAH activation gives rise to displacement of the autoregulatory region away from the catalytic domain, resulting in a more accessible catalytic site [22]. In our truncated PAHd form (Gly103-Thr427), Trp 120 is close to the N-terminal of the protein. Actually, the X-ray structure [14] does not show residues 103–117, which appear to be in a disordered conformation, and depicts Trp120 as exposed to the solvent, unlike in the structure of the full-length PAH construction [13]. This may be the reason why the tryptophan emission maximum of PAHd in native conditions is similar to that of the L-Phe activated full-length enzyme [15,18]. Interestingly, the spectroscopic similarity between PAHd and Phe-activated PAHt is retained along the urea denaturation process. The denaturation curves obtained for PAHt and PAHd (Figure S1) monitoring the I_355_/I_337_ fluorescence intensity ratio closely mimic those previously reported for non Phe-activated and Phe-activated full length PAH [11].

Urea-induced denaturation curves (Figure 3) for full-length PAHt and truncated PAHd were obtained following tryptophan fluorescence intensity at 345 nm (excitation at 295 nm). Two distinct transitions are evident, as previously reported for the full length protein [11]. The curves were thus fitted to a three-state unfolding model (Equation (2)). The model adequately describes the observed fluorescence intensity changes at 345 nm. However, given the rather complex aggregation landscape that appears to exist at intermediate urea concentrations (see AUC analysis below), no dissociation or aggregation events between subunits are contemplated in this three-state model. Besides, aggregation at high urea concentration compromises the reversibility of unfolding. Therefore, the values obtained in the fitting for the different parameters (Table 1) are not expected to closely reflect the true values of the corresponding magnitudes but should be regarded as an approximation. The three-state model constitutes therefore a qualitative description of the unfolding equilibrium.

As the stability of oligomeric proteins such as PAH is usually protein concentration dependent, urea equilibrium unfolding curves were obtained for PAHt and PAHd at two different protein concentrations. The effect of protein concentration on the fitted parameters are used to propose a qualitative model of the urea-induced unfolding equilibrium of PAHd and PAHt, which is then tested by AUC analysis.

At 0.8 µM subunit concentration (Figure 3a), two distinct transitions are observed for either form of PAH. In the initial transition, tryptophan fluorescence intensity increases by about 90% and 20% for PAHt and PAHd, respectively. This first transition is accompanied by a 2 nm red-shift for PAHt and a 7 nm blue-shift for PAHd. The apparent thermodynamic denaturation parameters of both constructions exhibit similar midtransition urea concentration values of 2.70 M for PAHt and 2.59 M for PAHd (Table 1). At higher urea concentrations, a second transition signaled by a decrease in fluorescence intensity and a 23 nm and 16 nm red-shift takes place in PAHt and PAHd forms, respectively. In this second step, the mid-transition urea concentration of PAHt is 0.5 M bigger than that of PAHd (5.48 M and 4.97 M, respectively).

To identify if any of the two transitions observed in either PAHt or PAHd may consist of or include associative or dissociative processes of the constituent subunits, the urea-induced denaturation profiles of the two forms were obtained at a higher subunit concentration. Thus, unfolding curves of 8 µM complete PAHt and truncated PAHd were recorded (Figure 3b), the curves fitted to a three-state model and their thermodynamic parameters compared to those at 0.8 µM concentration in Table 1.

Of the two parameters directly obtained using Equation (1) for each of the two transitions observed in the curves shown in Figure 3, the mid transition urea concentration, [urea]_m_, is by far the one that is more accurately calculated from urea unfolding curves analyzed with the LEM approach [23]. We center, therefore, our discussion on this parameter. Changing protein concentration has a clear effect on the mid-transition urea concentrations of the PAHd construction. The large difference of U_m1_ values at 0.8 and 8.0 µM (from 2.59 to 4.20 M urea) indicates that a dissociation event is taking place, which for PAHd can only consist of of its dissociation into monomers. Less clear is the reason why U_m2_ is at least moderately increased at this higher concentration. Both the fluorescence emission spectrum (describing the intermediate as not having the tryptophan residues fully exposed to solvent: Figure 2) and the ultracentrifugation analysis (see Section 2.2) appear to indicate that the monomers liberated in the first transition are not fully unfolded and seem to form aggregates at the urea concentrations where they accumulate. Those novel associations of monomers in the form of aggregates seem to break down in the second transition, which is thus also protein concentration-dependent (but not as much as U_m1_), yielding fully unfolded monomers (as judged by the emission spectrum, Figure 2b). In contrast, the complete PAHt construction shows essentially the same midtransition urea concentration for the first transition (U_m1_) irrespective of protein concentration. That no dissociation occurs in the first transition, together with the fact that the fluorescence of the PAHt intermediate resembles that of the PAHd intermediate, suggests that the tetramerization domain in PAHt impedes, at moderate urea concentrations, the dissociation of the otherwise partly unfolded catalytic domains that in PAHd dissociate in the equivalent first transition. As for the smaller but still clear increase in the U_m2_ of PAHt at the higher protein concentration, the reason may be similar to that proposed for PAHd, i.e., that the partly unfolded enzymatic domains can establish unspecific interactions among them that are broken in the second transition yielding fully unfolded enzymatic domains. Alternatively, the second transition in PAHt may additionally break the tetramerization interactions leading to the final dissociation of the four subunits.

**Figure 4:**
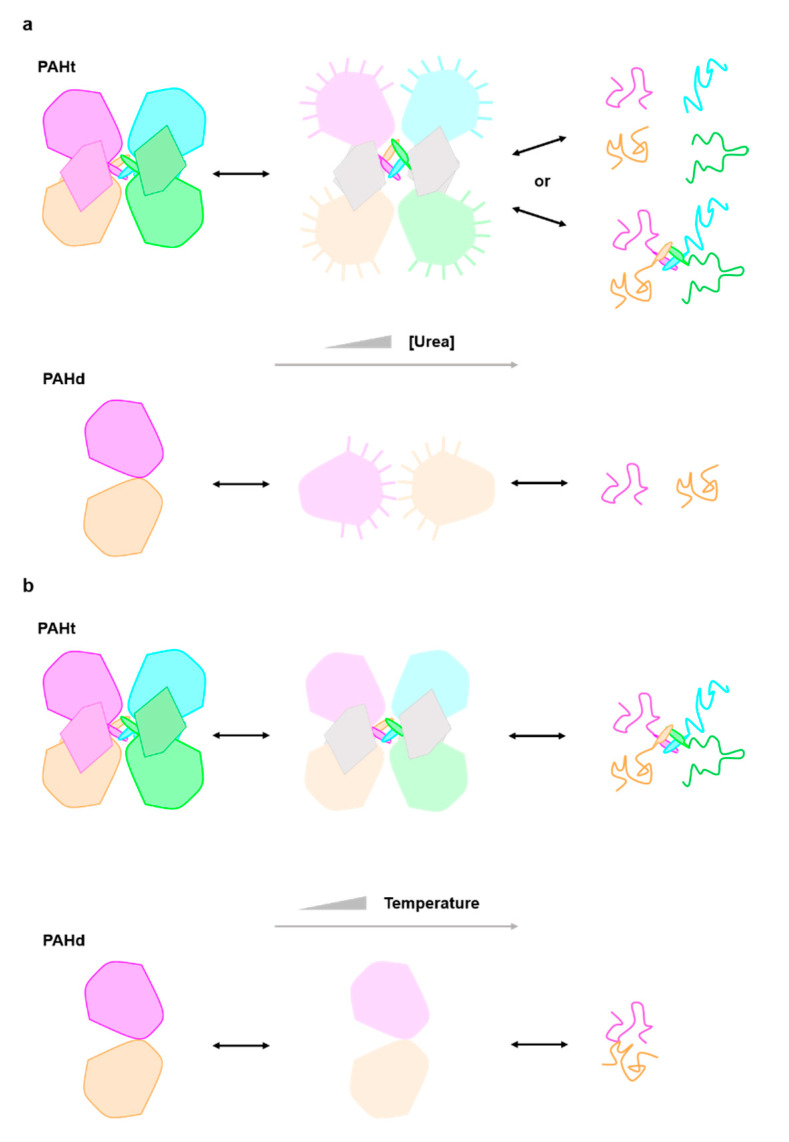
Structural cartoon describing the PAH three-state (**a**) chemical (urea) and (**b**) thermal unfolding equilibrium. Each subunit in PAHt is differently colored (dark pink, blue, orange and green) representing the four monomers in PAHt. Within each subunit, the large globule represents the catalytic domain and the small irregular pentagon the regulatory domain. The tetramerization helix of each monomer is also represented. The two subunits in PAHd (colored in dark pink and orange) are also represented by globules, as they consist essentially of the catalytic domain (see Figure 1). In the chemical unfolding driven by urea (**a**) the catalytic domains partly unfold (light color large globules) and remain in compact conformations where the tryptophan residues are not fully exposed to solvent. No information is available relative to the conformation of the regulatory domains (small irregular pentagons depicted in grey) in the intermediate. The interactions between pairs of catalytic domains in PAHt may be lost, but they can establish unspecific interactions with the same domains in other PAHt molecules, which is represented by lines pointing out from the potentially sticky surfaces in the partly unfolded catalytic domains. In the second step, the catalytic (and probably also the regulatory domains) unfold. The monomers may or may not dissociate. With the same color code, the urea unfolding equilibrium of PAHd is represented in the lower line. In PAHd, the two monomers dissociate in the intermediate, but their conformation appears to be compact, as judged by fluorescence data, and they seem to establish non-native interactions with additional monomers which are broken in the second step leading to the fully unfolded state. During thermal unfolding (**b**) the catalytic domains partly unfold but remain associated. No information is available relative to the conformation of the regulatory domains in the intermediate. In the second step, the catalytic (and probably also the regulatory domains) unfold. The monomers remain associated. The thermal unfolding equilibrium of PAHd is represented in the lower line. In PAHd, the two partly unfolded monomers remain associated in the intermediate and also in the fully unfolded state.

A previous work [11] proposed that in the first transition unfolding or rearrangement of the C-terminal helix occurred, transforming the tetrameric structure into a mixture of dimers and some monomers. However, our data reveal that the transition is also observed in the PAHd double truncated form where neither the N-terminal (regulatory) or the C-terminal (helix) domain are present. We thus propose a new model for the chemical denaturation pathway (Figure 4a) where the first step sees the partial unfolding of the catalytic domains, which may or may not remain associated in a tetrameric structure through interactions of the C-terminal helices. The partially unfolded catalytic domains are able to drive significant inter or intramolecular aggregation, and the second transition converts the aggregates into fully denatured monomers (which may or may not remain associated through their C-terminal helices). Whether the partial unfolding of the catalytic domain in PAHt concomitantly affects the folding of the regulatory domain cannot be assessed from the available data.

### 2.2. Analytical Ultracentrifugation Analysis of Urea-Induced Denaturation

**Figure 5:**
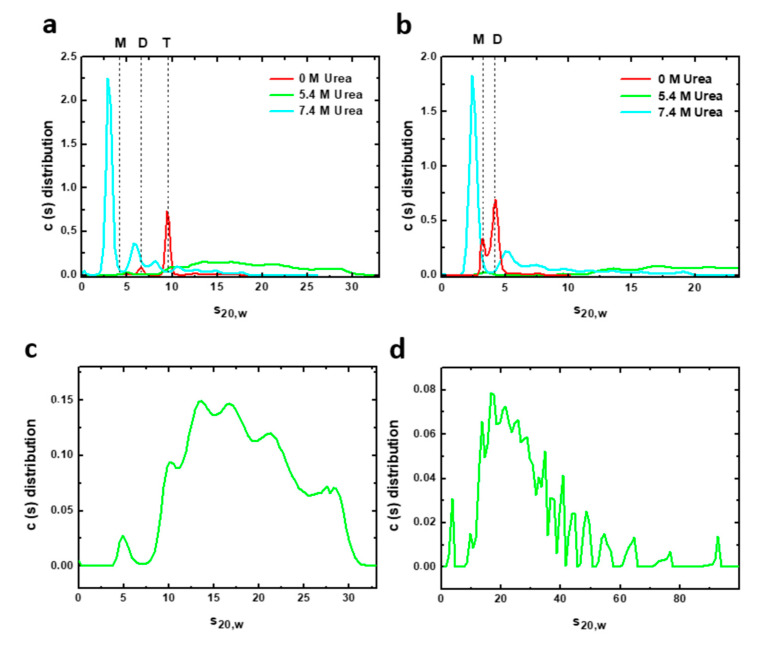
Velocity sedimentation (AUC) assays of human PAH in native and chemical denaturing conditions. Sedimentation profiles of (**a**) 8 µM PAHt and (**b**) 8 µM PAHd incubated at different urea concentrations: 0, 5.4 and 7.4 M. The solid lines represent the size distribution of sedimenting species obtained by c(s) analysis. Vertical discontinuous lines indicate the normalized sedimentation coefficients in water at 20 °C of different oligomeric PAH forms: tetramer (T), dimer (D) and monomer (M) in native conditions. Bottom panels are the sedimentation profiles of (**c**) 8 µM PAHt and (**d**) 8 µM PAHd incubated at 5.4 M urea obtained by c(s) analysis. PAH samples at this urea concentration are characterized by a great polidispersity with multiple species of fast sedimentation.

AUC experiments can complement spectroscopic information and help to clarify the oligomeric state of the potentially heterogeneous mixture appearing in the urea denaturation transitions. We therefore carried out AUC measurements of sedimentation velocities of both constructions under urea denaturing conditions (Figure 5). Sedimentation velocity analysis of the complete PAHt form in the absence of urea (Figure 5a) shows a predominant species responsible for 81% of the total protein signal with a sedimentation coefficient (s_20, w_) of 9.6 S, which corresponds to the tetramer. The frictional ratio is 1.27, consistent with a globular compact protein. Two minor species can be observed at 6.5 S and 12.8 S that can be attributed to a dimer (12% total signal) and aggregates (6% total), respectively. At 5.4 M urea, the PAHt sample shows a great polidispersity with multiple species of fast sedimentation at a broad range of 10.2–27.9 S representing 98% of the total protein signal (Figure 5c). Increasing the concentration of urea to 7.4 M (Figure 5a) gives rise to a major species at 3.1 S (62% total signal) which seems to correspond to a denatured monomer whose high frictional ratio of 1.58, indicative of a more extended conformation, suggests it is unfolded. Two minor species at 8.2 S and 6.0 S are additionally observed and attributed, respectively, to a denatured tetramer (8% total signal) and to a partially denatured dimer, or other species, with similar sedimentation properties (17% total signal). The rest of the protein signal at this high urea concentration, 13%, corresponds to aggregates of fast sedimentation in a range of 10.8–17.6 S formed by association of partially or totally denatured species.

The distributions here obtained at different urea concentrations by AUC can be compared with those reported in a previous urea-induced PAH denaturation experiment [11] monitored by size exclusion chromatography (SEC). The dissociation and denaturation model presented in [11] is partly consistent with the AUC data here reported. However, the AUC data reveal a more complex scenario as they clearly identify fast sedimentation aggregates formed by association of partially denatured species, which were not identified in the SEC experiments. Besides, at 7.4 M urea, AUC experiments identify denatured monomers along with denatured tetramers and denatured dimers, whereas in the SEC experiments only denatured monomers were observed at this urea concentration.

The double truncated PAHd form was also analyzed by AUC. In native conditions (Figure 5b), PAHd partitions into two major species at 4.2 S and 3.3 S, which correlates with native dimers and monomers accounting for 70% and 26% of the total protein signal, respectively. Two additional minor species with s_20, w_ of 7.5 S and 6 S are attributed to different aggregates of the truncated enzyme. At 5.4 M urea, PAHd shows a great polydispersity (Figure 5d) with multiple fast sedimentation species covering a very wide range of sedimentation coefficients (s_20, w_) from 9.6 S to 92.4 S and accounting for 98.3% of the total protein signal. This polydispersity, which is also observed in PAHt incubated at 5.4 M urea, seems characteristic of PAH chemical denaturation. The very large distribution of sedimentation coefficients indicates the presence of rapid dynamic association/dissociation processes at this urea concentration. Rapid sedimentation species represent aggregates formed by association of partially denatured species. At 7.4 M urea, however, PAHd shows a major peak at 2.5 S accounting for 60% of the total signal. This peak is ascribed to a monomer with a high frictional ratio of 1.57, indicating that it is an extended form of the monomer corresponding to a denatured species. The remaining 40% of the signal are species with sedimentation coefficients from 5.4 S to 18.8 S representing aggregates from association of different partially or totally denatured species.

Thus, velocity sedimentation experiments of complete PAHt and truncated PAHd constructions allowed us to analyze with greater resolution the composition of the initial, 0 M urea, and final, 7.4 M urea solution conditions of the urea unfolding curves previously monitored by fluorescence. These data are very useful to test the validity of the chemical unfolding model proposed in Figure 4a which was based only on fluorescence data. According to the AUC analysis done, the dominant starting conformations of PAHt and PAHd in absence of denaturant are the expected folded tetramers (81%) and dimers (70%), respectively. Those conformations are already in equilibrium with folded dimers (12%), in the case of PAHt, or folded monomers (26%) in the case of PAHd. Besides, small populations of aggregates are also present for either construction. At 7.4 M urea (the highest urea concentration analyzed) the dominant conformation (62%) of PAHt corresponds to a denatured monomer that, nevertheless, coexists with 17% of partly denatured dimers, 8% of denatured tetramers and 13% of fast sedimentation aggregates. Incomplete unfolding of the enzyme was previously reported by Kleppe R. et al. [11]. Our analysis also reveals that the dominant conformation (60%) of PAHd at this high urea concentration corresponds to denatured monomer, with the remaining 40% being aggregates. AUC data thus indicate that the two alternative main species proposed for the chemically unfolded state of PAHt (unfolded monomer and tetramer, Figure 4a, upper panel) actually coexist. The AUC data also confirm that the main species in the PAHd unfolded state is the unfolded monomer (Figure 4a, lower panel). Importantly, we were able to obtain a detailed view on the composition of the PAH protein solution at 5.4 M urea. Figure 3 indicates that at this urea concentration and at the protein concentration used in the AUC analysis (8 µM subunit), PAHt, is mostly (90%) in the macroscopic intermediate conformation defined by the three-state analysis, while PAHd is close to being 100% in the analogous macroscopic intermediate conformation. The AUC data at 5.4 M urea are thus suitable for dissecting the microscopic composition of the intermediate states defined by the fluorescence unfolding curves (Figure 3). For both PAHt and PAHd, the AUC analysis clearly indicates that their corresponding intermediates are composed of a very large mixture of coexisting species of fast sedimentation. Comparison of the PAHt and PAHd profiles in Figure 5 suggest that the tendency of PAHd to generate high molecular weight aggregates is even higher than that of PAHt, in agreement with the greater percentage of aggregates observed at 7.4 M urea for PAHd compared to PAHt. Although the *m* values annotated in Table 1 cannot be very accurate due to the poor reversibility of the curves, we noticed that the PAHd *m* values greatly increase with concentration, which is also in agreement with the PAHd intermediate having a greater tendency to form aggregates that the PAHt intermediate.

Overall, the AUC analysis is in qualitative agreement with the simplified model based in the fluorescence data (Figure 4a) but it emphasizes the microscopic complexity of the three macroscopic ensembles (native, intermediate and denatured). In this respect, it is clear that the native ensembles of PAHt and PAHd already contain some dimers or monomers, respectively, that the macroscopic intermediate is a polydisperse mixture of aggregated species, and that the denatured ensemble of PAHt, unlike that of PAHd contains, in addition to unfolded monomers, partly unfolded dimers and tetramers that likely remain associated by means of the oligomerization helices.

### 2.3. Fluorescence Characterization in Thermal-Induced Denaturation

**Figure 6:**
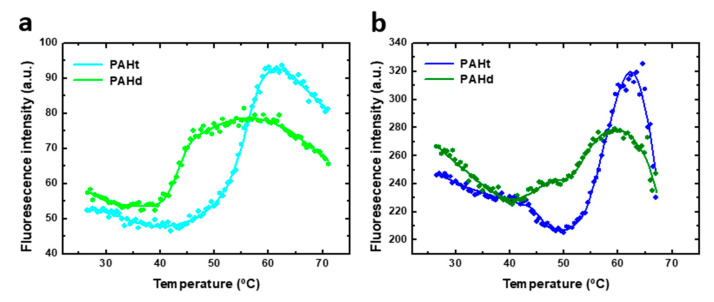
Thermal-induced denaturation of human PAH protein at different protein concentrations. Denaturation was monitored by fluorescence intensity at 345 nm (excitation at 295 nm) of (**a**) 0.8 µM of full-length PAHt construction and truncated PAHd construction, and (**b**) 5 µM of PAHt and PAHd. Continuous lines correspond to the nonlinear fitting curve obtained for a three-states folding model. Reported midtransition temperature values (T_m1_ and T_m2_) correspond to the average of individual nonlinear fittings of two or three thermal unfolding curves.

Dissection of denaturant-induced protein unfolding equilibrium of a protein may yield valuable information on the folding energetics and, in the case of oligomeric proteins, on the relative affinity among subunits. However, the stress induced on the protein conformation by chemical denaturants is not usually relevant in physiological conditions where thermal stability and, therefore, the study of the thermal-induced unfolding equilibria may be of greater interest. Trying to understand the PAH unfolding equilibrium as governed by temperature, we studied the thermal unfolding of human PAH using the PAHt and PAHd constructions at two different protein concentrations, as done before for the chemical denaturation assays. The subunit concentrations used were 0.8 µM and 5 µM. Higher protein concentrations could not be used as they give rise to aggregation during the heating (not shown). Thermal denaturation of both full-length PAHt and truncated PAHd forms was monitored by recording tryptophan fluorescence emission intensities at 345 nm (excitation at 295 nm). The unfolding curves of each construction (Figure 6) show two transitions at both protein concentrations used (0.8 µM and 5 µM subunit concentration), clearly revealing the accumulation of a folding intermediate. Thus, the thermal unfolding curves were fitted to a three-state unfolding model using Equations (2)–(4).

As indicated for the chemical denaturation experiments, the parameters values derived from the fitting of the thermal unfolding curves may not accurately reflect those of the corresponding thermodynamic magnitudes, and they should be considered as an approximation. Indeed, the steep baselines at high temperatures for the 5 µM curves may indicate that some aggregation is taking place at high temperature in this condition. Of the parameters obtained from the three-state fitting, the T_m_s and, particularly, T_m_1 are considered to be the more accurately determined ones. No enthalpy of heat capacity changes are reported because they are not considered accurate enough. The unfolding transitions of PAHd at 0.8 µM occur with T_m1_ and T_m2_ of 43.1 ± 0.2 °C and 54.1 ± 0.3 °C, respectively, which coincide with those obtained at 5 µM (43.6 ± 0.2 °C and 54.1 ± 0.3 °C, respectively). The unfolding transitions of PAHt at 0.8 µM subunit concentration occur with T_m1_ and T_m2_ of 46.8 ± 0.1 °C and 56.1 ± 0.7 °C, respectively, which also coincide with those obtained at the 5 µM subunit concentration (46.7 ± 0.2 °C and 56.6 ± 0.1 °C, respectively). The PAHt T_m_ values are thus higher than those of PAHd by about 3.5 °C and 2.5 °C, respectively. Unlike in the chemical denaturation of PAHd, and possibly also of PAHt, no dissociative or associative processes seem to be taking place in the thermal unfolding of either PAHt or PAHd. Therefore, the thermal unfolding intermediates of PAHd and PAHt do not seem to be the same as those accumulating in the chemical denaturation equilibrium. Unfolding intermediates populated along different unfolding pathways of a protein differ is not unprecedented. Differences in the number or nature of intermediate species present in the chemical and thermal unfolding equilibria have been described before, even for much simpler monomeric proteins [24,25,26]. A detailed AUC analysis of the thermal unfolding equilibrium is beyond the scope and capability of this work. Some structural information related to the transitions can be inferred, nevertheless, from the similar values of the PAHt and PAHd T_m_s, and from the spectroscopic similarities of the corresponding transitions. While the well-known unspecific effect of increasing temperature in decreasing fluorescence intensity can be observed in Figure 6 for the native baselines, the two transitions in the two constructions take place with a concomitant increase in fluorescence emission intensity. This means that, as seen in the first transitions of the chemical denaturation curves (Figure 3), the thermal transitions progressively reduce the initial quenching of tryptophan residues in the native starting conformations. That the T_m_s in PAHt are only slightly higher than those in PAHd, together with their similar spectroscopic behavior, suggest that they may correspond to transitions primarily affecting the catalytic domain (the only domain present in PAHd). The T_m_s of those transitions would then be modulated (shifted to slightly higher temperatures) by their context in the PAHt tetramer (i.e., by interactions of the catalytic domain with the regulatory domain or by the presence of the tetramerization helix).

Our results can be compared with previous thermal-induced denaturation studies of complete and truncated forms of the PAH enzyme [12,27]. A single transition was observed in thermal PAHt unfolding monitored by infrared spectroscopy [27]. This transition could correspond to the second transition described in our study since it showed a midtransition temperature value of 57 °C, similar to T_m2_ seen in Figure 6. The first transition may have not been detected by the infrared studies that were focused on changes in α-helix content. On the other hand, two transitions with T_m_ values of 46 °C and 54 °C were reported in a previous calorimetric study of full-length PAHt [12]. A truncated PAH (Asp112-Lys452) construct was also studied, and a single transition was observed with a T_m_ value similar to that reported here for T_m2_ of PAHt. A denaturation model with three stages was proposed after the analysis of DSC thermograms of full-length and truncated PAH forms [12]. The first step in thermal denaturation was proposed to be the unfolding of the four regulatory domains. After that, two of the four catalytic domains were denatured and, finally, irreversible protein denaturation accompanied by aggregates occurred. Since we detected in the thermal denaturation of PAHd, which lacks the regulatory domains, the same transitions experienced by PAHt, the first transition cannot be attributed to an unfolding event centered in the regulatory domains.

### 2.4. Differential Thermal Scanning in Thermal Unfolding

**Figure 7:**
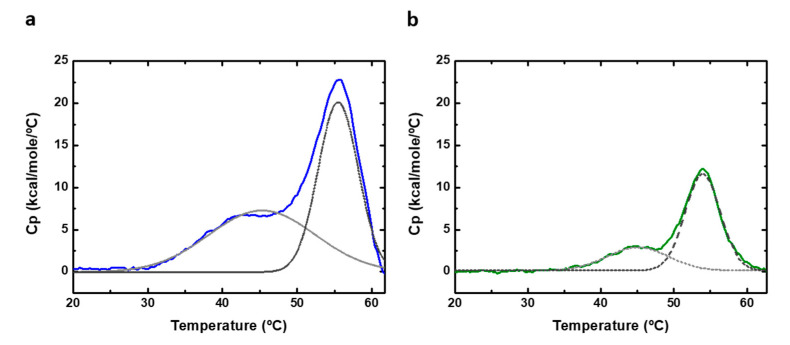
Differential scanning calorimetry (DSC) of human PAH. DSC thermograms of (**a**) 5 µM of full-length PAHt construction and (**b**) 5 µM of double truncated PAHd construction. Original profiles are normalized to protein concentration and the baseline subtracted. The excess heat capacity is shown. Dashed gray lanes correspond to deconvoluted peaks of DSC thermograms.

Differential scanning calorimetry (DSC) can be used to perform thermal denaturation studies. We recorded DSC scans of PAHt and PAHd (Figure 7) at the same subunit concentrations (0.8 and 5 µM) used in the thermal unfolding study followed by measuring fluorescence intensity. The obtained experimental heat capacity profiles are distorted at high temperatures, particularly at the higher concentration of 5 µM, as a consequence of aggregation, and they cannot be fitted to a theoretical three-state equilibrium model. No heat capacity or enthalpy changes can, therefore, be reported. Still, the DSC thermograms of both full-length PAHt and truncated PAHd show two clear, if partially overlapping, transitions (Figure 7). Their transition temperatures were approximately determined by local maxima calculation using the first derivate of the experimental profiles. For PAHt, the midtransition temperatures are 44 °C for the first transition and 55.6 °C for the second transition, similar to those obtained from the three-state fitting of the fluorescence unfolding curves in Figure 6b. The truncated PAHd construction presents midtransition temperatures of 45 °C and 54.4 °C, which are also in agreement with those more accurately determined from the fluorescence curves. No protein concentration dependency was found, in agreement with previous DSC studies [12].

The existence of an initial, low temperature transition observed in our work for the PAHd construction lacking the regulatory domain is in contrast with the denaturation model proposed in ref. [12] and with the data obtained in a later work [28]. As proposed above and illustrated in Figure 4b, the first transition observed in the double PAHd truncated construction could correspond to an equivalent unfolding event experienced by the full-length enzyme, which would take place at the catalytic domain, and that cannot be attributed to the unfolding of the regulatory domain. Alternatively, the first transition observed in PAHd in this work and not reported in [12] could be related to differences in experimental conditions. In this work, unlike in previous reported thermal studies [12,27,28], no stoichiometric amounts of Fe (II) were added to the recombinant protein to try to ensure that all the Fe(II) binding sites contain the iron ion. However, we reported previously the crystal structure of PAHd, using the same expression and purification protocol, and a full occupancy was observed for the cation.

### 2.5. Implications of the Thermal Unfolding Mechanism Proposed for the Interpretations of PAH Thermal Unfolding Curves as a Tool to Discover Novel Pharmacological Chaperones

That PKU, a rare disease that is primarily related to PAH destabilizing mutations [29], can be successfully treated in some cases by oral administration of the tetrahydrobiopterin PAH cofactor, whose complexation with the protein increases its thermostability [6,7], has stimulated the search for novel PKU pharmacological chaperones (PC) [8]. These are small organic compounds that stabilize the native conformation of an unstable PAH mutant thereby increasing the PAH native molar fraction and the effective activity.

In the search for novel PCs for a well characterized protein target such as PAH, miniaturized high throughput thermal unfolding assays [30], sometimes called thermofluor assays [31] or differential scanning fluorimetry [32], can be very useful and rapid techniques to identify potential leads. In the first of such screening projects performed to identify novel PCs for PAH [33], a small library of 1000 diverse compounds was screened by recording the thermal unfolding of PAHt in the presence of those compounds. To record the PAH thermal unfolding curves, a fluorescence signal was measured associated with the binding of an external probe, ANS, to hydrophobic surfaces of the protein arising as denaturation took place. Such curves, monitored by an external probe constitute an easy, general way to obtain an unfolding temperature that is a proxy for the real temperature(s) of mid-denaturation (T_m_) of the protein. In the indicated screening, the ANS-based unfolding curves were individually fitted to simplified equations, as described previously [33]. For four hits found in the screening, ANS-based thermal unfolding curves were subsequently recorded at different concentrations of the hits. In those curves two transitions analogous to those characterized in this work in the absence of ANS could be seen, although the curves were fitted to a two-state unfolding model in order to get a more robust, if less detailed, quantification of the concentration dependency of the stabilizing effect. A close inspection of the compounds’ concentration-dependent effect (Figure 2 in ref. [33]) indicates that they clearly stabilize the second transition but probably not so much the initial lower temperature transition. This scenario resembles that of the general protein engineering stabilization problem posed by proteins with a three-state thermal unfolding equilibrium [25,26,34]. For such proteins, stabilization of the second higher temperature transition (T_m2_) doesn’t protect the protein from unfolding at the lower temperature (T_m1_), which signals its loss of activity [24]. It has been demonstrated that specific increase of the lower denaturation temperature (T_m1_) is required and suffices to increase the relevant stability of the protein [35,36,37]. As thermal unfolding continues being a convenient way to screen for novel PC for PKU, the stabilizing effect of PC found in novel screening experiments should be analyzed, paying attention to the three-state macroscopic character of the PAH thermal equilibrium. If the two transitions observed in thermal unfolding monitored using external probes [33] mimic those observed and characterized here using intrinsic tryptophan fluorescence emission, the fact that the initial transition probably alters the native conformation of the catalytic domain suggests, based on equilibrium thermodynamic considerations, that PKU PCs increasing the first transition temperature (T_m1_) might be preferred over those only increasing the second temperature (T_m2_). This recommendation, however, doesn’t need to apply to PCs that exert their effect in a kinetic (speeding up the folding reaction) rather than in a thermodynamic manner (stabilizing the natively folded enzyme molecules against unfolding), or that combine the two mechanisms [38].

## 3. Materials and Methods

### 3.1. Protein Expression and Purification

Expression of recombinant full-length human PAH construction (PAHt) and double truncated form (PAHd) and their purification were performed as described in [38]. PAHt and PAHd were expressed as maltose-binding fusion proteins in *E. coli* BL21 (DE3) cells grown in Super Broth medium with 100 µg/mL of ampicillin at 37 °C. Expression was induced by 1 mM IPTG, and the culture was prolonged at 20 °C for two days. Recombinant MBP-PAH proteins were purified using amylose affinity chromatography followed by gel filtration on Superdex 75 XK26/60. Collected fractions were cleaved overnight with PreScission Protease at 4 °C and the untagged proteins were further purified using two affinity columns, MBPTrap HP and GSTrap 4B (GE Healthcare). A second size exclusion chromatography step was then used to isolate the tetrameric form of PAHt and the dimeric form of PAHd. The proteins were concentrated and their final concentrations were determined spectrophotometrically using theoretical extinction coefficients at 280 nm [39].

### 3.2. Fluorescence Urea Denaturation Measurements

The two protein constructions (at 0.8 µM and 8 µM subunit concentrations) were incubated overnight, in darkness, at 25 °C, in 20 mM Tris/HCl, 200 mM NaCl, pH 7.4 in presence of 0–7.8 M urea. Tryptophan emission spectra of equilibrium denatured samples at 25 °C were registered (excitation at 295 nm; slit 5 nm), from 300 to 460 nm on a Cary Eclipse Fluorescence Spectrophotometer (Varian). Unfolding curves represented by the fluorescence intensity at 345 nm were analyzed using Equation (1), which corresponds to a three-state unfolding equilibrium [23,40,41]:

(1) $$F=\frac{\left(F_{N}^{0}+m_{N}\times D\right)+\left(F_{I}^{0}+m_{I}\times D\right)\times e^{-\left(m_{1}\times \left(U_{m1}-D\right)\right)/RT}+\left(F_{U}^{0}+m_{U}\times D\right)\times e^{-\left[\left(m_{1}\times \left(U_{m1}-D\right)\right)+\left(m_{2}\times \left(U_{m2}-D\right)\right)\right]/RT}}{1+e^{-\left(m_{1}\times \left(U_{m1}-D\right)\right)/RT}+e^{-\left[\left(m_{1}\times \left(U_{m1}-D\right)\right)+\left(m_{2}\times \left(U_{m2}-D\right)\right)\right]/RT}}$$

where *F* corresponds to the fluorescence intensity signal at 345 nm, *D* is the denaturant concentration, $F_{N}^{0}$, $F_{I}^{0}$ and $F_{U}^{0}$ represent the fluorescence signal of the native, intermediate and unfolded protein in absence of denaturant, and $m_{N}$, $m_{I}$ and $m_{D}$ are the slopes of the linear dependency with denaturant concentration attributed to the corresponding fluorescence signals. Furthermore, *R* is the universal gas constant and *T* is the absolute temperature. $U_{m1}$ and $U_{m2}$ correspond, respectively, to the midtransition urea concentration for the first (from native to intermediate state) and second (from intermediate state to unfolded state) transition, and $m_{1}$ and $m_{2}$ to the slopes of the linear dependencies with denaturant concentration of the first and second transitions’ folding free energies.

### 3.3. Velocity Sedimentation Experiments by Analytical Ultracentrifugation

Sedimentation velocity measurements were performed at 25 °C and 45,000 rpm using a Beckman-Coulter Optima XL-I analytical ultracentrifuge equipped with absorbance and Rayleigh interference optics. Protein samples (8 µM subunit concentration) were incubated in their respective urea solutions overnight before the start of the sedimentation velocity experiments. The sedimentation coefficients were calculated from c(s) size distribution analysis implemented in SEDFIT 14.7 g [42]. The solvent density, viscosity and partial specific volume of the proteins were calculated using the SEDNTERP program [43] (http://www.jphilo.mailway.com/download.htm, accessed 16 June 2021) and, thus, the sedimentation coefficients were corrected to standard conditions of water and 20 °C (s20,w). Extra hydrodynamic analyses with SEDNTERP were done to ascribe a consistent oligomeric state for the main peaks of AUC profiles and to calculate their frictional ratios [44].

### 3.4. Fluorescence Thermal Denaturation Measurements

Full-length and double truncated PAH thermal denaturation was followed, from 20 to 90 °C, by fluorescence emission intensity. Unfolding curves were acquired at 345 nm, exciting samples at 295 nm, and using a heating rate of 1 °C·min^−1^. A range of protein concentrations (0.8–10 µM) of both constructions were prepared for unfolding in 20 mM Tris, 200 mM NaCl at pH 7.4. Each individual unfolding curve was fitted to a three-state model, as described in [23], using Equation (2):

(2) $$F=\frac{\left(F_{N}^{0}+m_{N}\times T\right)+\left(F_{I}^{0}+m_{I}\times T\right)\times e^{-\Delta G_{1}/RT}+\left(F_{U}^{0}+m_{U}\times T\right)\times e^{-\left(\Delta G_{1}+\Delta G_{2}\right)/RT}}{1+e^{-\Delta G_{1}/RT}+e^{-\left(\Delta G_{1}+\Delta G_{2}\right)/RT}}$$

where $\Delta G_{1}$ and $\Delta G_{2}$, are the unfolding Gibbs energy changes for the native to intermediate transition and for the intermediate to denatured transition, respectively, as given by Equations (3) and (4)

(3) $$\Delta G_{1}\left(T\right)=\Delta H_{1}^{T_{m1}}\times \left(1-T/T_{m1})+{\Delta C}_{p1}\times [T-T_{m1}-T\times \ln(T/T_{m1})\right]$$

(4) $$\Delta G_{2}\left(T\right)=\Delta H_{2}^{T_{m2}}\times \left(1-T/T_{m2})+{\Delta C}_{p2}\times [T-T_{m2}-T\times \ln(T/T_{m2})\right]$$

In the previous equations, *F* is the observed fluorescence intensity signal at a given temperature while $\left(F_{N}^{0}+m_{N}\times T\right)$, $\left(F_{I}^{0}+m_{I}\times T\right)$ and $\left(F_{U}^{0}+m_{U}\times T\right)$ represent the temperature dependent signal of either the native, the intermediate or the unfolded state of the protein, respectively. In Equations (3) and (4), *T_m_*, Δ*H*, and Δ*C_p_* are the temperature of mid denaturation, the unfolding enthalpy change, and the unfolding heat capacity change of the indicated transition.

### 3.5. Differential Scanning Calorimetry (DSC)

Differential scanning calorimetry (DSC) assays were performed at 5 µM PAHt or PAHd (subunit concentration) in 20 mM Tris/HCl, 200 mM NaCl, pH 7.4. Measurements were performed on a VP-DSC micocalorimeter (MicroCal LLC, Northampton, MA) from 10 to 90 °C at a scanning rate of 1 °C·min^−1^. The reference and sample solutions were degassed and carefully loaded into the calorimetric cells to avoid bubble formation. Baselines were recorded before each assay with the reference and sample cells filled with working buffer (20 mM Tris/HCl, 200 mM NaCl, pH 7.4). The baselines were subtracted from the DSC protein thermograms, which were then normalized to protein concentration using the software package Origin (OriginLab). The midtransition temperature values were calculated from the first derivative of the thermograms.

## 4. Conclusions

PAH chemical and thermal unfolding equilibria can be described by a three-state model. In the corresponding intermediate conformations, which in chemical denaturation consists of a polydisperse ensemble of aggregates, the catalytic domain is unfolded and appears to be compact, as judged by their fluorescence properties. The structural disruption of the catalytic domain in the first (lower temperature) thermal unfolding transition suggests that those PCs for PKU that specifically stabilize PAH variants against the first unfolding transition should be more efficacious, other things being equal, than those that only stabilize the enzyme against its full unfolding taking place at higher temperatures.

## Figures and Tables

**Table 1:** Apparent thermodynamic parameters of urea-induced unfolding equilibrium of the human PAH protein. Reported values of m and [urea]m correspond to the result of global nonlinear fitting to a three-state model of two or three chemical unfolding curves for each construction at the different protein concentrations indicated. The errors indicated are those obtained in the global nonlinear fitting.

PAH Construction	[Subunit] (µM)	Transition	[Urea]m (M)	m (kcal·mol^−1^·M^−1^)
PAHt(1-452)	0.8	1st	2.70 ± 0.05	1.91 ± 0.24
		2nd	5.48 ± 0.11	2.12 ± 0.58
	8	1st	2.73 ± 0.08	1.23 ± 0.14
		2nd	6.38 ± 0.17	1.21 ± 0.17
PAHd(103-427)	0.8	1st	2.59 ± 0.11	2.37 ± 0.76
		2nd	4.97 ± 0.27	1.02 ± 0.47
	8	1st	4.20 ± 0.03	4.61 ± 1.09
		2nd	6.54 ± 0.13	2.47 ± 0.59

## Supplementary Materials

The following are available online at https://www.mdpi.com/article/10.3390/ijms22126539/s1.

## Abbreviations

ANS, 8-Anilinonaphthalene-1-sulfonic acid; AUC, analytical ultracentrifugation; DSC, differential scanning calorimetry; GST, glutathione S-transferase; IPTG, isopropyl β-D-thiogalactoside; MBP, maltose binding protein; PAH, human phenylalanine hydroxylase; PAHt, the full-length human PAH (Met1-Lys452); PAHd, a double truncated human PAH form (ΔN1-102/ΔC428-452); PC, pharmacological chaperone; PKU, phenylketonuria
