# Supplementary material for: Unravelling the Complex Denaturant and Thermal-Induced Unfolding Equilibria of Human Phenylalanine Hydroxylase
Source: Int J Mol Sci. 2021 Jun 18;22(12):6539. doi: 10.3390/ijms22126539 (PMC8234983; doi:10.3390/ijms22126539)
Supplement: Supplementary file 1 [file ijms-22-06539-s001.zip › ijms-1255969-supplementary.pdf]

Supplementary material

Unravelling the complex denaturant- and thermal-induced unfolding equilibria of human phenylalanine hydroxylase

María Conde-Giménez and Javier Sancho

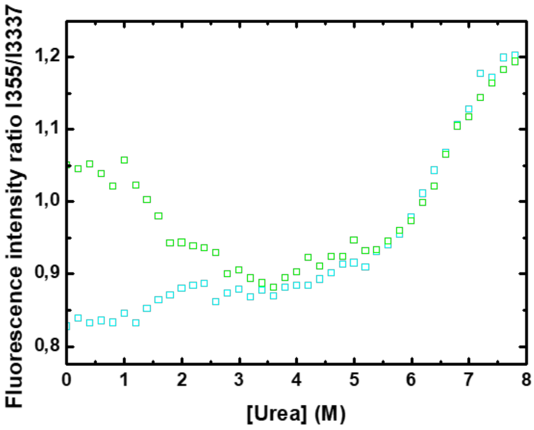

Figure S1. Urea-induced denaturation of human PAH protein. Unfolding was monitored by the ratio of fluorescence intensities at 355 nm and 337 nm (I_{355}/I_{337}) of 0.8 µM PAHt (in cyan) and 0.8 µM PAHd (in green).
